# Baroreflex sensitivity following acute upper-body exercise in the cold among stable coronary artery disease patients

**DOI:** 10.3389/fphys.2023.1184378

**Published:** 2023-10-13

**Authors:** Kalle Pikkarainen, Rasmus I. P. Valtonen, Heidi E. Hintsala, Antti Kiviniemi, Craig G. Crandall, Juha Perkiömäki, Arto J. Hautala, Mikko P. Tulppo, Jouni J. K. Jaakkola, Tiina M. Ikäheimo

**Affiliations:** ^1^ Center for Environmental and Respiratory Health Research, Research Unit of Population Health, University of Oulu, Oulu, Finland; ^2^ Research Unit of Biomedicine and Internal Medicine, Medical Research Center Oulu, Oulu University Hospital, University of Oulu, Oulu, Finland; ^3^ Centria University of Applied Sciences, Kokkola, Finland; ^4^ Department of Internal Medicine, Texas Health Presbyterian Hospital, Institute for Exercise and Environmental Medicine, University of Texas Southwestern Medical Center, Dallas, TX, United States; ^5^ Faculty of Sport and Health Sciences, University of Jyväskylä, Jyväskylä, Finland; ^6^ Medical Research Center, University of Oulu, Oulu University Hospital, Oulu, Finland; ^7^ Department of Community Medicine, UiT The Arctic University of Norway, Tromsø, Norway

**Keywords:** autonomic nervous system, baroreflex, blood pressure variability, cold, exercise, upper-body exercise, coronary artery disease

## Abstract

**Background:** A cold environment and exercise separately affect the autonomic nervous system (ANS), baroreflex sensitivity (BRS), and blood pressure variability (BPV) but their combined effects on post-exercise recovery are not known. Our cross-over trial examined these responses following upper-body static and dynamic exercise performed in a cold and neutral environment in patients with coronary artery disease (CAD).

**Methods:** 20 patients with stable coronary artery disease performed both graded static (10%–30% of maximal voluntary contraction) and dynamic (light, moderate and high perceived intensity) upper-body exercise at −15°C and +22°C for 30 min. Electrocardiogram and continuous blood pressure were measured to compute post-exercise (10 and 30 min after exercise) spectral powers of heart rate (HR), blood pressure variability and BRS at low (0.04–0.15 Hz) and high (0.15–0.4 Hz) frequencies.

**Results:** Static upper-body exercise performed in a cold environment increased post-exercise high frequency (HF) spectral power of heart rate (HF RR) (*p* < 0.001) and reduced heart rate (*p* = 0.001) and low-to-high frequency (LF/HF) ratio (*p* = 0.006) more than in a neutral environment. In addition, post-exercise mean BRS (*p* = 0.015) and high frequency BRS (*p* = 0.041) increased more following static exercise in the cold than in a neutral environment. Dynamic upper-body exercise performed in a cold environment reduced post-exercise HF BRS (*p* = 0.019) and systolic blood pressure (*p* = 0.003).

**Conclusion:** Static upper-body exercise in the cold increased post-exercise BRS and overall vagal activity but without reduced systolic blood pressure. Dynamic upper-body exercise in the cold reduced post-exercise vagal BRS but did not affect the other parameters. The influence of cold exposure on post-exercise autonomic and cardiovascular responses following static upper-body exercise require further studies. This information helps understanding why persons with cardiovascular diseases are vulnerable to low environmental temperature. ClinicalTrials.gov: NCT02855905 (04/08/2016).

## 1 Introduction

Cold temperatures globally increase all-cause mortality with most of the excess mortality caused by moderately cold temperatures (Gasparrini et al., 2015). Low environmental temperatures affect the cardiovascular system and often results in adverse cardiovascular outcomes (Ikäheimo, 2018), such as higher morbidity and mortality (Fares, 2013; Liu et al., 2015). Wintertime physical activity may also be a common trigger of sudden cardiac deaths (Toukola et al., 2015). These outcomes are more common among individuals with ischemic heart disease. Cold exposure augments cardiovascular strain manifested as increased heart rate (HR) by 5–10 bpm and systolic blood pressure (SBP) by 15–20 mmHg in healthy persons as well as in individuals with coronary artery disease (CAD) (Manou-Stathopoulou et al., 2015; Ikäheimo, 2018). The higher cardiac workload of cold exposure leads to higher myocardial oxygen demand (Manou-Stathopoulou et al., 2015). Furthermore, an imbalance between oxygen demand and supply can cause myocardial ischemia and provoke angina.

The baroreflex is the dominant short-term blood pressure regulation mechanism, which changes blood pressure by altering the activation of vagal cardioinhibitory neurons to the heart and sympathetic neurons to heart and peripheral blood vessels (La Rovere et al., 2008). Baroreflex sensitivity (BRS) represents the autonomic neural regulation of the cardiovascular system and can be evaluated in many ways by assessing the variations in HR and blood pressure (BP). Impaired baroreflex sensitivity is often associated with cardiovascular diseases, including CAD (Katsube et al., 1996). BRS is lowered among CAD patients, correlating with the severity of coronary artery narrowing (Katsube et al., 1996). Furthermore, low BRS and heart rate variability (HRV) are associated with increased cardiac mortality after myocardial infarction, and low BRS and HRV combined increase the risk even further (La Rovere et al., 1998).

During exercise, the baroreflex is reset to operate at a higher level of blood pressure depending of the intensity of the exercise while maintaining its sensitivity (Raven et al., 2019). BRS is decreased after exercise and recovers to pre-exercise levels in 30–60 min, depending on the intensity and type of exercise (Niemelä et al., 2008). Both aerobic and resistance exercise lower BRS, but the effect is greater during resistance exercise (Heffernan et al., 2007). HRV is reduced and blood pressure variability (BPV) is elevated during post-exercise recovery, suggesting decreased vagal activity and augmented sympathetic activity (Niemelä et al., 2008).

Cold exposure increases BRS among healthy persons at rest, as demonstrated during a cold face test (Eckberg et al., 1984; Hilz et al., 1999; Stemper et al., 2002) as well as during cold exposure without cooling the face (Yamazaki and Sone, 2000; 2001; Mourot et al., 2007). Hintsala et al. (2016) also demonstrated that cold exposure augmented BRS among hypertensive patients. To our knowledge, there are no previous studies describing the influence of whole-body cold exposure on upper-body exercise-related BRS responses. Furthermore, it is possible that the different modes of exercise (dynamic and static) influence post-exercise BRS differentially.

The aim of this study was to assess the effects of static and dynamic upper-body exercise in a cold environment on indices of post-exercise autonomic function, BRS and BPV. We tested the hypothesis that cold exposure may blunt the known post-exercise withdrawal of sympathetic activity (and reduction in BRS), with this occurring to a greater extent after static exercise. We conducted our study among CAD patients who might have a higher risk for cardiovascular events during the cold season.

## 2 Materials and methods

### 2.1 Subjects

The study design, recruitment and intervention protocols, and thermal measurements have also been described previously (Valtonen et al., 2022). The study consisted of 20 male patients with stable CAD. The selected number of participants is based on a sample size estimation and power analysis (Kang, 2021) for a prior study (Hintsala et al., 2014) that indicated statistically significant differences in BP between baseline and after cold exposure [Power (1-ß err prob), 0.9, Cohen’s effect size 0.8, *α* err prob 0.05] would be expected with 15 participants. To account for any unexpected increases in variability, we chose to assess 20 patients. The patient recruitment took place 10–12/2015 and 10–12/2016 and the controlled measurements were performed at Kastelli Research Center Oulu, Finland. The study participants were recruited from the group of patients who had been treated recently at the Oulu University Hospital and at least 3 months after a non-ST-elevation myocardial infarction. During their hospital stay, the patients underwent thorough examinations including echocardiography. The selection of patients was conducted by a cardiologist who utilized available clinical data and interviews to confirm the suitability of the participants. To avoid confounding factors in the study experiments, some predetermined inclusion criteria were set. These consisted of diagnosed CAD (Canadian Cardiac Society [CCS] class I–II, a non-ST-elevation myocardial infarction occurring at least 3 months ago, male sex, age range from 35 to 75 years, left ventricular ejection fraction ≥40%, no known or suspected atrial fibrillation, claudication, asthma or diabetes, and no electrocardiographic changes, such as bundle branch block, significant intraventricular conduction delay, or ST-segment/T-wave changes. Patients with a pacemaker or a history of coronary artery bypass grafting were not included in the study, neither were current smokers since smoking has adverse cardiovascular influences Clinical exercise tests were performed to assess physical capacity and to detect possible ECG abnormalities (ST-segment depression over 3 mm, three consecutive premature ventricular contractions). For this purpose, bicycle ergometer tests (Ergoline, ergoselect 100K, Fysioline, Finland) were carried out approximately a month prior to the experiments. Prior to the test, ECG and HR were measured at rest in the supine position. The test started from 30 W and was increased by 15 W each minute until exhaustion. An exercise physiologist carried out the tests, which were monitored by a medical doctor. Peak oxygen consumption was estimated using the formula: 3.5 × MET, where MET is metabolic equivalent. Peak MET values were gathered from the maximal clinical exercise testing procedure. Prior to the experiments, body composition was assessed by bioimpedance measurements (InBody720 Biospace, Seoul, Korea) and from where we obtained % body fat and weight for calculating BMI. While attending the experiments the subjects also completed a questionnaire inquiring about their perceived health (excellent, quite good, average, quite poor), as well as health behaviors, such as the degree of physical strain at work (mainly sitting, lot of walking, lot of walking and lifting of items, heavy manual labor) and frequency of strenuous leisure time activities (never, rarely, often, very often), perceived physical fitness (excellent, quite good, average, quite poor) and any use of alcohol (%), as well as current medication (Table 1). Details of the studied patients are presented in Table 1 and in our previous publication from the same population (Valtonen et al., 2022). They were given both oral and written information about the study and an informed consent was required for participation. The study was conducted in accordance with the declaration of Helsinki and was approved by the Ethics Committee of the Northern Ostrobothnia Hospital District, Oulu, Finland. All patients provided informed consent to participate to the study. The study was registered at ClinicalTrials.gov (NCT02855905, 04/08/2016).

**TABLE 1:** Characteristics of the study population of CAD patients (n = 20).

Variables	Mean (SD) or n (%)
Age, years	59 (8.5)
Body mass index, kg/m2	28 (3.9)
Body fat, %	24 (6.0)
Estimated peak oxygen consumption, mL/kg/min	31 (5.5)
Systolic blood pressure, mmHg	120 (13)
Diastolic blood pressure, mmHg	77 (11)
Hypertension, n	18 (90%)
Time from MI, months	29 (11)
Single vessel disease, n	13 (65%)
Double vessel disease, n	5 (25%)
Triple vessel disease, n	2 (10%)
Number of stents, n	2 (0–4)
Left ventricular ejection fraction	63% (8.5)
Medication	
*Acetylsalicylic acid, n*	20 (100%)
*Beta-blockers, n*	15 (75%)
*Statins, n*	16 (80%)
*Angiotensin converting enzyme inhibitors, n*	8 (40%)
*Angiotensin receptor blockers, n*	6 (30%)
*Calcium channel blockers, n*	3 (15%)
Pensioner, n	12 (60%)
Perceived health, n	
*Excellent*	4 (20%)
*Quite good*	8 (40%)
*Average*	7 (35%)
*Quite poor/Very poor*	1 (5%)
Any use of alcohol	19 (95%)
Physical demands of work, n	
*Mainly sitting*	13 (65%)
*A lot of walking*	2 (10%)
*A lot of walking and lifting*	5 (25%)
*Heavy manual labor*	0 (0%)
Leisure-time physical activity, n	
*Never*	1 (5%)
*Rarely*	12 (60%)
*Often*	5 (25%)
*Very often*	1 (5%)
Physical fitness status, n	
*Excellent*	3 (15%)
*Quite good*	9 (45%)
*Average*	7 (35%)
*Quite poor*	1 (5%)

The values represent the number of the patients or means ± SD., peak oxygen consumption, in mL/kg/min, was estimated (3.5*MET, where MET, is metabolic equivalent of task) from a symptom-limited maximal oxygen uptake test.

### 2.2 Applied upper-body exercise

Each patient (n = 20) participated in four different interventions in a climatic chamber, with each intervention performed in random order. These interventions were static upper-body exercise at cold (−15°C) and neutral (+22°C) conditions, and dynamic upper-body exercise in the same conditions, each for 30 min. While being exposed to cold, the patients wore full winter clothing consisting of underwear (shirt, pants), insulated trousers, insulated jacket, overtrousers, overjacket, socks and shoes (insulation value of clothing ensemble 2.13 clo). This clothing ensemble was selected to replicate the clothing one would wear in the winter months. The static upper-body exercise consisted of 5-min pre-exposure followed by five 1.5-min static contraction against a bench press with different intensities (Newtest Leg Force [bench press mode], Newtest, Oulu, Finland), performed at 10%, 15%, 20%, 25% and 30% of maximal voluntary contraction (MVC). MVC was determined as the largest of three bench press contractions aiming to maximal force and measured approximately 1 hour before the first trial. Participants had a 4-min break following each session. The dynamic upper-body exercise sessions consisted of 5-min pre-exposure followed by three 5-min exercise periods of arm cranking exercise (Monark 881E, Vansbro, Sweden) at different intensities. The intensity of exercise was adjusted to mild-, moderate and high and based on subjective ratings of Perceived Exertion (11–12 fairly light as a mild-, 13–14 somewhat hard as a moderate- and 15–16 hard as a high-intensity), with the identical workloads performed between thermal conditions. Subjects had 4 min rest between each dynamic exercise bout.

The post-exercise measurements of ANS function and BRS were conducted at 10 min and 30 min following the exercise in a climatic chamber which temperatures was set to +22°C.

### 2.3 Autonomic nervous system function and baroreflex sensitivity

Brachial systolic and diastolic BP was recorded with oscillometric sphygmomanometry from the left arm (Schiller BP 200 Plus; Schiller, Baar, Switzerland). We supported the arm to place the cuff (for arm circumference of 25–35 cm) approximately at the level of the heart. Rate-pressure product (RPP) was computed as systolic BP x HR to estimate cardiac workload.

We examined continuous arterial BP with a non-invasive method that applies the volume clamp principle (Nexfin, BMEYE Medical systems, Netherlands). The BP cuff (size according to measured finger circumference) was placed at the forefinger of the right hand, and the arm was supported approximately at the level of the heart. A height sensor was placed at the level of the heart to allow the Nexfin device to automatically correct for the hydrostatic pressure influences. Respiration was recorded with a piezo-electric belt (PneumoTrace, ADInstruments, Australia). Continuous BP, respiratory and ECG signals were connected to the laptop with an analog-to-digital transformer with 1 kHz sampling frequency (Power Lab/8SP, ADInstruments, Australia) managed with Labchart software (v7.3.2, ADInstuments, Australia). These signals were recorded for 5 min at each of the following periods: baseline, 10 min post-exercise, and 30 min post-exercise. We are aware that HF-power, and other vagally related HRV-measures, might be confounded by different breathing patterns (Grossman and Taylor, 2007). Such effects were minimized through controlled measurements performed with patients in the seated position and instructed to avoid talking, and yawning, while breathing normally during the measurements. The researchers further monitored the respiration signal and registered only the data that contained stable breathing patterns. Data were measured once before each intervention and twice (5–10 min and 25–30 min) during recovery measurements. We performed data analyses for the recorded continuous BP, respiratory and ECG data with a custom-made Matlab-based (MathWorks, Inc., Natick, MA, United States) software. Ectopic or anomalous beats were removed from the signals manually and replaced with local average. Data sequences missing upon automatic calibration of the Nexfin were replaced with a linear interpolation method. Time series of RR-interval and beat-to-beat systolic BP were extracted. We estimated total BP variability as a standard deviation of systolic BP. For the spectral analyses, very low frequencies (<0.04 Hz) were detrended by the Savitzky-Golay method. We then computed spectral estimates for stabilized periods of baseline and cold exposure (5 min from the end of both phases) on LF (0.04–0.15 Hz) and HF (0.15–0.4 Hz) bands of HR and BP variability by applying FFT (Welch’s method, sequence length 128, overlapping 50%). Spectral estimate of spontaneous BRS was computed on LF band with alpha method as follows *BRS = √HRV_LF_/BPV_LF_, in which HRV_LF_ and BPV_LF_* denote RR-interval variability and systolic BP variability on LF band. The method presumes a high degree of linear correlation of RR-interval and systolic BP, and therefore analyses were done only if the coherence between HR and BP variability was >0.5. Respiration frequency was computed manually as the amount of respiration cycles/time.

### 2.4 Skin temperature

Skin temperature was measured continuously using thermistors (NTC DC95, Digi-Key, Thief River Falls, MN, United States) attached to the right scapula, left cheek, forehead, left calf, right anterior thigh, dorsal side of left index finger (middle phalanx), left hand, left forearm, right shoulder, left upper chest. Data were recorded at 20 s intervals with two temperature data loggers (SmartReaderPlus; Acr Systems Inc., BC, Canada). Mean skin temperature (Tsk) was calculated as follows: tsk = ∑ki*tski = [0.07*forehead+0.175*right scapula+0.175*left upper chest+0.07*right arm+0.07*left arm+0.05*left hand+0.19*right anterior thigh+0.2*left calf] (ISO 9886). Thermal sensations were obtained using scales of perceptual judgements on personal thermal state (ISO10551).

### 2.5 Statistical methods

Cardiovascular parameters with a non-Gaussian distribution were transformed into natural logarithm for parametric statistical tests. The differences in the means between neutral environment and cold, as well as the different time points (before, and 10 and 30 min after the intervention) and temperature*time interaction were compared by two-way repeated measures ANOVA. For any observed interaction, separate *post hoc* analyses were conducted to compare means between the temperature conditions (cold vs neutral), as well as post-exercise measurement point (baseline vs 10 min or 30 min). The results are presented as means with 95% confidence intervals. Statistical analyses were performed with IBM SPSS for.

Windows version 20 (IBM Corp. Released 2011; Armonk, NY, United States) and significance was set at *p* < 0.05.

## 3 Results

The characteristics of the study participants are presented in Table 1. Twelve of the patients with CAD were retired from employment, while the rest were still a part of working life. The physical fitness of the patients (estimated VO_2_ peak 30.0 ± 5.6 mL/kg/min) was on average moderately physically fit when using scales of healthy adults (Shvartz and Reibold, 1990) and where 45% rated their physical fitness as being quite good. However, 60% reported rarely being physically active during their leisure time. Most (95%) of the patients rated their health status as being moderate or better. The average time elapsed from the myocardial infarction was 29 months 65% of the patients had a single-vessel, 25% a double-vessel and 10% a triple-vessel disease and having on average two stents. The ejection fraction of the patients was on average 63%.

### 3.1 Static exercise in the cold

Exposure to cold temperature decreased mean skin temperature by 4.1°C (*p* < 0.001) and facial skin temperature from +31°C to +15°C (*p* < 0.001) by the end of exercise. At the end of the intervention, the average rating of whole-body thermal sensation was −2/cold (cold) and +1/slightly warm (neutral). Skin temperature recovered close to baseline values 30 min post cold exposure, with mean skin temperature being just 1°C and facial skin temperature being 2°C–3°C lower after exposure to the cold condition (Hintsala et al., 2021). The identical graded workloads represented 46, 47, 48, 52% and 56% of HR_max_ at neutral environment and 42, 43, 44, 47% and 50% in a cold environment, with HR_max_ values derived from the incremental leg exercise test. The RPE during exercise varied from fairly light to very hard (10–16) for both temperatures.

When examining post-exercise responses, both temperature and time (post-exercise measurement point) independently affected several parameters of ANS, BPV and BRS (Table 2). In addition, when examining their interaction, acute static upper-body exercise performed in a cold environment increased post-exercise HF RR (*p* < 0.001) and reduced HR (*p* = 0.001) and LF/HF ratio (*p* = 0.006) more than in a neutral environment. In addition, post-exercise mean BRS (*p* = 0.015) and HF BRS (*p* = 0.041) increased more following static exercise in the cold than in a neutral environment. Most of the ANS, BRS and BPV parameters remained elevated compared with baseline during the 30 min follow-up.

**TABLE 2:** Post exercise responses to static exercise in neutral and cold environment among CAD patients (n = 20).

Variable	Exercise neutral (22°C)			Exercise cold (−15°C)			*p*-values		
	Pre-exercise	10 min	30 min	Pre-exercise	10 min	30 min	T	t	T × *t*
HR, bpm	66 (60, 72)	63 (58, 68)^*^	64 (59, 69)	64 (61, 67)	58 (55, 61)^*#^	60 (57, 63)^*#^	**0.004**	**< 0.001**	**0.001**
LF RR, ms^2^	421 (224, 619)	453 (236, 670)	543 (260, 826)^*^	518 (230, 806)	435 (227, 642)	767 (243, 1,292)^*^	0.167	**0.004**	0.696
HF RR, ms^2^	166 (60, 272)	223 (62, 384)	213 (51, 374)	202 (79, 325)	314 (181, 447)^*#^	334 (184, 483)^*#^	**0.002**	**< 0.001**	**< 0.001**
LF/HF	4.0 (1.7, 6.3)	3.4 (1.2, 5.6)	3.9 (1.9, 5.9)	4.1 (1.7, 6.5)	1.7 (1.0, 2.4)^*#^	2.8 (1.6, 4.1)^*#^	**0.011**	**0.001**	**0.006**
									
SBP, mmHg	120 (112, 127)	123 (114, 132)	123 (114, 132)	120 (112, 128)	128 (121, 135)^*^	128 (121, 135)^*^	0.200	**0.023**	0.300
DBP, mmHg	76 (70, 81)	78 (73, 84)	78 (73,83)	75 (70, 80)	79 (75, 84)^*^	79 (73, 85)^*^	0.940	**0.003**	0.444
LF BPV, mmHg^2^	11.2 (3.9, 18.6)	8.6 (4.5, 12.8)	14.1 (6.3, 22.0)^*^	10.1 (3.7, 16.5)	4.6 (2.8, 6.5)^#^	9.0 (4.2, 13.8)^#^	**0.002**	**< 0.001**	0.134
HF BPV, mmHg^2^	2.8 (2.2, 3.3)	2.9 (2.1, 3.7)	3.5 (2.8, 4.2)	3.8 (2.6, 5.0)	3.5 (2.2, 4.7)	4.0 (3.0, 4.9)	0.864	0.187	0.911
									
LF BRS, ms/mmHg	7.1 (5.4, 8.8)	7.4 (5.8, 9.1)	6.0 (4.9, 7.2)	7.7 (6.1, 9.3)	9.7 (7.9, 11.6)^#^	9.3 (7.6, 11.0)^#^	**0.001**	**0.036**	0.104
HF BRS, ms/mmHg	7.2 (5.1, 9.2)	8.6 (5.7, 11.4)	7.0 (5.0, 9.0)	8.0 (5.5, 10.5)	11.4 (7.8, 15.0)^*#^	9.8 (7.0, 12.5)^*#^	**0.009**	**0.001**	**0.041**
Mean BRS, ms/mmHg	7.1 (5.5, 8.7)	8.0 (5.9, 10.2)	6.5 (5.0, 8.0)	7.9 (5.9, 9.8)	10.6 (8.1, 13.0)^*#^	9.5 (7.5, 11.5)^*#^	**0.001**	**0.001**	**0.015**

Values are group means and 95% confidence intervals. *HR*, heart rate; *LF*, low frequency; *HF*, high frequency, *RR*, interval; *BP*, blood pressure; *SBP*, systolic blood pressure; *DBP*, diastolic blood pressure, *BPV* (systolic) blood pressure variability, *BRS*, baroreflex sensitivity. *p*-values are for interaction effects of two-way repeated measures ANOVA: T temperature (cold or neutral), t time (baseline, 10 min post exposure or 30 min post exposure).

^*^ *p* < 0.05 vs baseline (time), # < 0.05 vs. the respective trial in the neutral condition (temperature).

The bolded values denote statistically significant differences (*p* < 0.05).

### 3.2 Dynamic exercise in the cold

Exposure to cold decreased mean skin temperature by 3.7°C (*p* < 0.001) and facial skin temperature decreased considerably from +31°C to +15°C (*p* < 0.001) during dynamic exercise in the cold environment. At the end of the intervention, the average whole-body thermal sensation was −1/slightly cool (cold) and +2/warm (neutral). Post-intervention skin temperature recovered close to the baseline after 30 min from cold exposure, with mean skin temperature being 1°C and facial skin temperature being 2°C–3°C lower after exposure to cold conditions. The identical pedalling speeds kept during exercise at both temperatures represented 56, 62% and 73% of HR_max_ in a neutral and 59, 66% and 80% of HR_max_ in a cold environment, with HR_max_ values derived from the incremental leg exercise test. The RPE reported at the highest workload during dynamic exercise varied from fairly light to hard (11–15) at the neutral temperature and from somewhat hard to very hard (12–16) in the cold environment.

In contrast to static exercise, temperature did not affect post-exercise ANS, BPV or BRS (Table 3). Instead, altered responses were observed mainly when comparing the post-exercise measurement point with baseline. Only post-exercise HF BRS (*p* = 0.019) and systolic BP (*p* = 0.003) were lowered more compared with baseline after exercise in the cold. These parameters remained elevated during the follow-up compared with baseline.

**TABLE 3:** Post-exercise responses of dynamic upper-body exercise in neutral and cold environment among CAD-patients (n = 20).

Variable	Exercise neutral (22°C)			Exercise cold (−15°C)			*p*-values		
	Pre-exercise	10 min	30 min	Pre-exercise	10 min	30 min	T	t	T × *t*
HR, bpm	62 (59, 65)	71 (68, 75)^*^	70 (66, 74)^*^	62 (59, 65)	73 (69, 77)^*^	70 (66, 74)^*^	0.599	**< 0.001**	0.123
LF RR, ms^2^	471 (198, 743)	239 (90, 388)^*^	328 (134, 523)^*^	469 (201, 738)	216 (70, 363)^*^	330 (135, 524)^*^	0.871	**< 0.001**	0.336
HF RR, ms^2^	278 (98, 458)	88 (38, 138)^*^	149 (58, 240)^*^	269 (125, 412)	94 (27, 162)^*^	118 (32, 204)^*^	0.522	**< 0.001**	0.093
LF/HF	2.8 (1.6, 4.0)	4.1 (2.2, 5.9)^*^	3.4 (2.0, 4.8)	2.5 (1.6, 3.3)	4.5 (2.2, 6.8)^*^	4.3 (2.7, 5.8)^*^	0.635	**< 0.001**	0.212
									
SBP, mmHg	118 (109, 128)	117 (110, 124)	119 (112, 126)	123 (114, 131)	114 (107, 122)^*#^	114 (107, 120)^*#^	0.797	0.055	**0.003**
DBP mmHg	74 (68, 80)	77 (72, 82)	75 (70, 80)	73 (68, 79)	73 (68, 78)	74 (69, 79)	0.452	0.607	0.258
LF BPV, mmHg^2^	6.6 (4.2, 9.0)	6.0 (3.3, 8.8)	8.9 (5.8, 12.0)^*^	10.4 (4.7, 16.1)	6.7 (2.7, 10.7)^*^	9.5 (4.8, 14.3)	0.923	**0.001**	0.973
HF BPV, mmHg^2^	3.5 (2.4, 4.5)	2.9 (1.9, 3.9)	4.1 (2.7, 5.5)	3.6 (2.5, 4.7)	3.1 (2.5, 3.8)	3.8 (2.9, 4.8)	0.244	0.054	0.165
									
LF BRS, ms/mmHg	7.4 (5.7, 9.1)	5.6 (4.4, 6.9)	5.5 (4.3, 6.7)^*^	7.6 (5.9, 9.2)	5.9 (4.0, 7.7)^*^	5.7 (4.1, 7.4)^*^	0.976	**< 0.001**	0.470
HF BRS, ms/mmHg	9.4 (5.4, 13.4)	6.4 (3.2, 9.6)^*^	6.7 (3.9, 9.5)^*^	9.9 (5.8, 14.0)	5.4 (2.4, 8.4)^*#^	5.6 (2.9, 8.3)^*#^	0.263	**< 0.001**	**0.019**
Mean BRS, ms/mmHg	8.4 (5.7, 11.1)	6.0 (3.9, 8.1)^*^	6.1 (4.2, 8.0)^*^	8.7 (6.2, 11.2)	5.6 (3.3, 8.0)^*^	5.7 (3.7, 7.7)^*^	0.370	**< 0.001**	0.064

Values are group means and 95% confidence intervals. *HR*, heart rate; *LF*, low frequency; *HF*, high frequency, *RR*, interval; *BP*, blood pressure; *SBP*, systolic blood pressure; *DBP*, diastolic blood pressure, *BPV* (systolic) blood pressure variability, *BRS*, baroreflex sensitivity. *p*-values are for interaction effects of two-way repeated measures ANOVA: T temperature (cold or neutral), t time (baseline, 10 min post exposure or 30 min post exposure).

* *p* < 0.05 vs. baseline (time).

# *p* < 0.05 vs. the respective trial in the neutral condition.

The bolded values denote statistically significant differences (*p* < 0.05).

## 4 Discussion

Our novel findings demonstrate that static upper-body exercise in the cold augments post-exercise HF RR and reduces HR and LF/HF ratio more than after exercise in a neutral environment. In addition, post-exercise baroreflex sensitivity (HF and mean BRS) is higher compared with the corresponding exercise in a neutral environment among patients with CAD. In contrast, the change in ANS, BRS or BPV following dynamic exercise did not differ between a cold and neutral environment, except for HF BRS, which was lowered more in cold compared with the neutral environment.

### 4.1 Previous findings related to the separate effects of cold exposure and acute exercise

The impact of cold exposure on BRS has earlier been restricted to studies conducted at rest and which have demonstrated augmented baroreflex sensitivity among healthy individuals using various forms of cold exposure, such as the cold face test (Eckberg et al., 1984; Hilz et al., 1999; Stemper et al., 2002), water-perfused suit (Yamazaki and Sone, 2000; 2001), cold water immersion (Mourot et al., 2007) and whole-body cold air exposure in a climatic chamber among hypertensive patients (Hintsala et al., 2016). These have involved in general very short (60–120 s) (Hilz et al., 1999; Stemper et al., 2002), short (4–5 min) (Yamazaki and Sone, 2000; Mourot et al., 2007; Hintsala et al., 2016) or somewhat longer (10 min) recordings (Eckberg et al., 1984) of HRV.

The increase of BRS in cold may be caused by either a direct central vagal activation (Stemper et al., 2002) or an interaction between baroreceptors and skin thermoreceptors (Eckberg et al., 1984; Yamazaki and Sone, 2000). Exposure to cold at rest causes sympathetic activation and increased BP which has also been shown to increase both HF and LF BPV among hypertensive and healthy subjects (Hintsala et al., 2016).

The arterial baroreflex is reset to defend lowered blood pressures after acute exercise in humans and results in reduced post-exercise sympathetic outflow (Halliwill et al., 2013). Recovery from exercise is also related with immediate postexercise hyperaemia and sustained postexercise vasodilatation reducing BP. At the same time cardiac output may remain higher due to increased HR. These post-exercise cardiovascular responses are related to the type and intensity of the exercise (Halliwill et al., 2013).

### 4.2 Static exercise in the cold

Static upper-body exercise involves sustained contraction that causes mechanical compression of both of muscles and vasculature, where the related ischemia and stimulation of metaboreceptors increases sympathetic activity and elevates blood pressure during this form exercise (Gonzalez-Camarena et al., 2000; Machado-Vidotti et al., 2014). The increased HR during static exercise, on the other hand, is thought to result from the influence of reduced parasympathetic activity to the sinus node (Iellamo et al., 1999). Our results concerning post-exercise responses of exercise in the cold showed significantly higher post-exercise HF power of heart rate variability (HF RR) which is accepted as a marker of vagal activity (Lombardi et al., 1996). Furthermore, a reduction in the LF/HF ratio could further indicate a shift to stronger vagal dominance. However, this response should be interpreted with some caution as LF/HF ratio has also been questioned as an indicator of sympatho-vagal balance (Bilman, 2013) We further showed augmented post-exercise BRS (HF BRS, mean BRS) supporting increased vagal activity. Post-exercise HR was also reduced and in line with these changes. LF BPV, which is considered as a surrogate measure of vascular sympathetic activity and regulation of the vascular tone (Julien, 2006; Stauss, 2007), was lowered after exercise, but with no interaction for temperature.

The observed augmented post-exercise vagal response is in line with the results of our previous study from the same population showing reduced HR that prevailed during the static exercise in a cold environment (Valtonen et al., 2022). There are a few possible mechanisms that may explain this finding. The cessation of static exercise involves continuously increased parasympathetic activity, perhaps due to a reduction in central command (Iellamo et al., 1999). In addition, cooling of the facial skin during exercise stimulates the trigeminal nerve and evokes a non-baroreflex mediated vagal response (Khurana and Wu, 2006) that can further prevail after exercise and influence the observed vagal response. Previous studies have also suggested that the cessation of static exercise leads to a rapid withdrawal of sympathetic activity (Martin et al., 1974; Niemelä et al., 2008). Though, the slightly increased post-exercise LF and elevated SBP and DBP observed in our study suggest some influence of increased sympathetic activity. Supporting this notion, our earlier study from the same population showed a lowered HR, but at the same time higher mean arterial pressure that was maintained throughout the static exercise in the cold (Valtonen et al., 2022). Furthermore, our previous study examining post-exercise central aortic BP responses concerning with static exercise showed a sustained higher BP indicative of sustained sympathetic activity and/or elevated cardiac output alongside the vagal activation (Hintsala et al., 2021).

Our study detected that post-exercise BRS remained elevated compared with baseline during the 30 min follow-up after static exercise in the cold. This could be related to the delayed recovery of skin temperature towards thermoneutral levels following exercise in the cold and possibly sustaining elevated cardiac vagal activity, supported by our observations of simultaneous lower HR, higher HF RR and lower LF/HF ratio. In contrast to our findings, BRS is usually reduced immediately after static exercise (in a neutral environment) and eventually returns to baseline values (Niemelä et al., 2008). Though, its recovery may be delayed after static exercise of higher intensities.

To our knowledge, there are only a few studies that examined cardiovascular responses related to static upper-body exercise in the cold. Excluding our recent research among CAD patients (Hintsala et al., 2021; Valtonen et al., 2022), the other studies have involved healthy persons. Koutnik et al. (2014) observed a higher central aortic BP with static handgrip exercise in the cold, but only included a 3-min post-exercise follow-up. Consistent with our findings Mäkinen et al. (2008) demonstrated that HR is lowered following isometric hand-grip exercise in a cold environment compared with a neutral environment. On the other hand, Greaney et al. (2014) did not find difference in changes of HR and BP following handgrip exercise between cold and neutral environment. The differing findings are likely due to the varying study protocols.

### 4.3 Dynamic exercise in the cold

Dynamic upper-body aerobic exercise involves cycles of muscular contraction and relaxation and increased blood flow to the working muscles, vasodilation and related cardiac volume loading (Manou-Stathopoulou et al., 2015). This mode of exercise has been shown to relate to a rapid withdrawal of vagal activity and where the concurrent increase in sympathetic activity contributes to higher BP and HR during exercise (Gonzalez-Camarena et al., 2000, Tulppo et al., 1999). Our study showed that dynamic upper-body exercise in a cold and neutral environment provided comparable post-exercise autonomic and BRS responses. Only HF BRS was lowered more following the exercise in cold compared with a neutral environment. The other BRS parameters showed a similar, but a non-significant decreasing trend. The decrease and resetting of BRS after acute exercise in a neutral environment have been shown previously following acute exercise among healthy persons (Niemelä et al., 2008) and those with CAD (Hanssen et al., 2022). The diminished post-exercise HF BRS with decreased HF RR after exercise in the cold suggests a reduction in cardiac vagal activity, probably related to the discontinuation of facial cooling. However, at the same time mean skin temperature remained approximately 1 °C lower compared with baseline which could to some extent sustain elevated sympathetic activity. This could explain the observed higher HR and LF/HF ratio compared to baseline.

A post-exercise lowering of SBP was observed which is consistent with previous studies related to acute exercise among healthy persons (Halliwill et al., 2013) and CAD patients (Kiviniemi et al., 2015). The reduction in SBP was more pronounced after exercise in the cold, perhaps indicative of a more pronounced withdrawal of sympathetic activity. However, the significance of this result remains unclear, as our previous study detected lowered central aortic systolic BP without interaction for temperature (Hintsala et al., 2021).

Only few studies have been conducted investigating the effects of upper-body exercise in cold of cardiac autonomic responses (Franklin et al., 1995; Smolander et al., 1995), but these did not focus on such responses compared with a neutral environment. To our knowledge, there are no previous studies assessed BRS in response to combined dynamic upper-body exercise and cold exposure.

### 4.4 Differential post-exercise recovery after static and dynamic upper-body exercise in the cold

Differences in the post-exercise effects on ANS and BRS of static *versus* dynamic exercise after cold exposure could be due to differential cardiovascular (pressure vs volume loading) and thermal responses of these modes of exercise.

Recovery after static exercise in the cold seems to be delayed, as judged by the elevated BRS, increased parasympathetic activity, and lowered HR. Such responses could be due to a few reasons. The increased parasympathetic activity during exercise coupled with the vagal response induced by facial cooling (Khurana and Wu, 2006) during the chamber exposure could continue also after cessation of the exercise and exiting the cooler climate. In addition, static exercise increases sympathetic activity due to the previously mentioned pressor response and muscle chemoreflex activation (Iellamo et al., 1999). Furthermore, whole-body cooling of the skin contributes to additional sympathetic activation and increases vascular resistance (Manou-Stathopoulou et al., 2015). Some of these responses may carry-over after the cessation of exercise and exiting the cooler conditions, resulting in heightened sympathetic activity that may blunt the known post-exercise reduction in BP (Hintsala et al., 2021).

The relatively similar and quick post-exercise recovery following dynamic exercise could be due to a few reasons. In contrast to static exercise, the increased blood flow to the working muscles and accompanying vasodilation could offset peripheral cold-induced vasoconstriction during exercise and enable quick post-exercise recovery (Hintsala et al., 2021). It is also possible that bradycardia related to facial cooling is dampened due to the increased sympathetic activity during dynamic exercise in the cold and would not influence post-exercise responses. Though, the exact mechanism for these differing post-exercise responses remains uncertain and requires further studies.

### 4.5 Applicability

Our results demonstrate differential post-exercise effects for static and dynamic upper-body exercise related to the regulation of ANS and BRS. Static exercise performed in a cold environment in CAD patients involves a heightened post-exercise vagal response and increased BRS. At the same time, no post-exercise reduction in SBP was observed. In contrast, post-exercise responses of dynamic upper-body exercise were not much influenced by temperature. The findings of this and our previous (Hintsala et al., 2021) study concerning recovery from static exercise after exercise in the cold calls for further studies to examine the neural and cardiovascular mechanisms in more detail.

The study produces new information explaining post-exercise mechanisms on autonomic nervous system function and showing that these can be altered by low environmental temperature. This could be used for planning individual exercise-based training programs for patients with CAD. The need to examine exercise in a cold environment among patients with a ischemic heart diseases relates to further understanding the potential mechanisms behind the observed higher occurrence of cardiovascular events during the cold season (Fares, 2013; Liu et al., 2015) and often connected with physical activity (Manou-Stathopoulou et al., 2015; Toukola et al., 2015; Ikäheimo, 2018).

The current research is also topical due to the changing climate and the expected higher share of weather extremes which render preparedness and protection challenging. Persons with ischemic heart diseases represent a climate sensitive population and are particularly vulnerable to climate change.

### 4.6 Strengths and limitations

The strengths of the study include a strictly controlled level of thermal exposure and exercise. Furthermore, each subject served as his own control; therefore, eliminating potential confounders due to interindividual factors. In addition, randomization of the trials limited an order effect. Finally, strict selection of participants helps reduce confounding variables from causes other than those related to cardiovascular diseases. The study involves some limitations as well. These data demonstrate the responses in patients with CAD, which is important to understand the associated risks specific to this population. However, we cannot exclude the possibility that similar responses would be observed in similarly aged individuals without CAD. For safety reasons, we did not cease medication of the patients during the experiments. Hence, we evaluated cardiovascular responses of individuals who are being treated for CAD, rather than examining the disease in the absence of medical treatment. As a limitation, including a longer follow-up could have brought further insight of BRS recovery. Finally, interpretation of HRV as reflecting sympathetic activity should be made with caution due to its interrelation with parasympathetic activity, as well as to possible mechanical effects of breathing and prevailing heart rate.

### 4.7 Conclusion

Our findings suggest that especially static upper-body exercise performed in a cold environment increases post-exercise vagal activity and baroreflex sensitivity among patients with stable CAD. In contrast, post-exercise responses of upper-body dynamic exercise were not influenced much by temperature. Connected to the changing climate, future research is needed for examining how different forms of exercise carried out both in cold and hot environments influences autonomic nervous system function, baroreflex sensitivity and blood pressure among persons with different cardiovascular diseases.

## Data Availability

The original contributions presented in the study are included in the article/Supplementary material, further inquiries can be directed to the corresponding author.
